# Mapping rapeseed (*Brassica napus L.*) aboveground biomass in different periods using optical and phenotypic metrics derived from UAV hyperspectral and RGB imagery

**DOI:** 10.3389/fpls.2024.1504119

**Published:** 2024-12-06

**Authors:** Chuanliang Sun, Weixin Zhang, Genping Zhao, Qian Wu, Wanjie Liang, Ni Ren, Hongxin Cao, Lidong Zou

**Affiliations:** ^1^ Department of Digital Technology, Institute of Agriculture Information, Jiangsu Academy of Agricultural Sciences, Nanjing, China; ^2^ School of Mathematical and Computational Sciences, Massey University, Auckland, New Zealand; ^3^ School of Computer Science and Technology, Guangdong University of Technology, Guangzhou, China; ^4^ Institute of Applied Artificial Intelligence of the Guangdong-Hongkong-Macao Greater Bay, Shenzhen, China; ^5^ School of Artificial Intelligence, Shenzhen Polytechnic University, Shenzhen, China

**Keywords:** rapeseed (*Brassica napus L.*), aboveground biomass (AGB), phenotypic metrics, hyperspectral images (HSI), machine learning approach

## Abstract

Aboveground biomass (AGB) is a key indicator of crop nutrition and growth status. Accurately and timely obtaining biomass information is essential for crop yield prediction in precision management systems. Remote sensing methods play a key role in monitoring crop biomass. However, the saturation effect makes it challenging for spectral indices to accurately reflect crop changes at higher biomass levels. It is well established that rapeseed biomass during different growth stages is closely related to phenotypic traits. This study aims to explore the potential of using optical and phenotypic metrics to estimate rapeseed AGB. Vegetation indices (VI), texture features (TF), and structural features (SF) were extracted from UAV hyperspectral and ultra-high-resolution RGB images to assess their correlation with rapeseed biomass at different growth stages. Deep neural network (DNN), random forest (RF), and support vector regression (SVR) were employed to estimate rapeseed AGB. We compared the accuracy of various feature combinations and evaluated model performance at different growth stages. The results indicated strong correlations between rapeseed AGB at the three growth stages and the corresponding indices. The estimation model incorporating VI, TF, and SF showed higher accuracy in estimating rapeseed AGB compared to models using individual feature sets. Furthermore, the DNN model (R^2^ = 0.878, RMSE = 447.02 kg/ha) with the combined features outperformed both the RF (R^2^ = 0.812, RMSE = 530.15 kg/ha) and SVR (R^2^ = 0.781, RMSE = 563.24 kg/ha) models. Among the growth stages, the bolting stage yielded slightly higher estimation accuracy than the seedling and early blossoming stages. The optimal model combined DNN with VI, TF, and SF features. These findings demonstrate that integrating hyperspectral and RGB data with advanced artificial intelligence models, particularly DNN, provides an effective approach for estimating rapeseed AGB.

## 1 Introduction

Winter oilseed rape (*Brassica napus L.*) is one of the most important oil crops globally, with China accounting for about one-third of the world’s cultivated area and one-fifth of total production (Liu et al., 2019). The Yangtze River basin is the primary growing region for this crop in China. In addition to providing essential oil products, cultivating oilseed rape offers benefits such as improving soil fertility and serving as a potential raw material for bioenergy. Therefore, efficiently and accurately monitoring rapeseed growth is crucial for enhancing both yield and quality. Timely estimation of AGB is particularly important for diagnosing nutrient deficiencies, guiding precise fertilization, and predicting yield outcomes.

AGB is a critical physiological indicator for monitoring crop growth and guiding agricultural management. It is closely linked to the crop’s nutritional status and the ability of leaves and stems to absorb organic matter, making it a key variable in crop phenotyping (Araus and Cairns, 2014). Accurate AGB monitoring is essential for effective crop management, yield prediction, and ensuring food security through a stable supply (Li et al., 2020, Li Z. et al., 2022). However, traditional methods of estimating AGB—such as destructive field sampling followed by laboratory drying and weighing—are time-consuming and inefficient. These approaches do not meet the need for large-scale, high-throughput, timely, and quantitative monitoring, thereby limiting real-time AGB assessment at the field scale (Chang et al., 2017; Yue et al., 2018b; Zeng et al., 2018).

In the past decade, research on crop growth monitoring using UAV-mounted spectral platforms has emerged as a new direction in precision agriculture. Hyperspectral imaging and UAV technology have significantly improved the flexibility of predicting crop AGB (Tao et al., 2020; Yue et al., 2020, 2023), enabling the collection of crop phenotypic information at the field scale for growth monitoring (Clevers et al., 2017; Gitelson et al., 2003; Kooistra and Clevers, 2016). Previous studies have successfully utilized these technologies for biomass monitoring in crops such as rice, maize, barley, wheat, and grasslands (Bendig et al., 2014, 2015; Derraz et al., 2023; Shu et al., 2023; Sinde-González et al., 2021; Zhai et al., 2023). Compared to UAV-mounted RGB and multispectral sensors, hyperspectral sensors cover a broader range of spectral bands, allowing for a deeper investigation of crop physiological characteristics (Daughtry et al., 2000). This technology has shown promising results in yield prediction for soybean and disease monitoring for wheat (Banerjee et al., 2020; Guo et al., 2021; Herrero-Huerta et al., 2020. Therefore, the application of spectral imaging technology is essential for accurately detecting spatial variability in crop biochemical composition.

Previous studies on the estimation of rapeseed AGB were based on the methods of index and texture characteristics, however there were few studies on the estimation of rapeseed biomass in combination with structural parameters. Current research on estimating AGB using optical data primarily focuses on leveraging vegetation indices (VI) that are sensitive to dry matter content in crop canopies (Cheng et al., 2017; Hansen and Schjoerring, 2003; Itoh et al., 2006). VI capture changes in crop physiological activity and canopy structure, facilitating accurate AGB estimation (Wang et al., 2019). Several studies have validated effective VI for estimating AGB in crops such as winter wheat, maize, rice, and cotton using traditional regression methods. Commonly used indices include the Normalized Difference Vegetation Index (NDVI), Visible Atmospherically Resistant Index (VARI), Transformed Vegetation Index (TVI), and Red-edge Chlorophyll Index (Han et al., 2019; Ma et al., 2022; Pugh et al., 2018; Varela et al., 2017). However, due to differences in canopy structure and growth stages across species, the performance of VI can vary, making AGB estimation less reliable at different growth stages. For instance, before canopy closure, interactions with soil background and spectral saturation can reduce the accuracy of VI-based AGB estimates (Yao et al., 2018; Yue et al., 2017, 2023).

High-resolution imagery provides rich texture features at the plot level. Previous studies have successfully used texture features derived from satellite data to estimate AGB, particularly in forested areas (Zha et al., 2020; Zhang et al., 2015). Additionally, AGB prediction can be achieved using 3D data from UAV sensors, which combine height information from LiDAR or stereo imagery with spectral features from multispectral images (Muharam et al., 2014; Zhu et al., 2019). These studies have demonstrated that texture and structural parameters significantly improve crop monitoring accuracy. However, the high cost and weight of LiDAR sensors make routine crop growth monitoring challenging. In contrast, RGB photogrammetric sensors are lighter and more practical, making them a viable alternative. Therefore, exploring the potential of extracting crop structural parameters from RGB data is crucial for crop monitoring. In recent years, the integration of artificial intelligence (AI) algorithms with optical data for AGB estimation has emerged as a promising new approach. AI methods are particularly advantageous for processing multi-dimensional data, effectively addressing the issue of data redundancy that is common in traditional regression models (Li R. et al., 2022; Verrelst et al., 2012; Volpato et al., 2021; Wang et al., 2016).

Currently, there is limited research on the accuracy of AGB estimation models for rapeseed using canopy spectral, texture, and structural information extracted from UAV-based hyperspectral and RGB images. To assess the potential of combining optical and phenotypic parameters for rapeseed AGB estimation, the objectives of this study are: (1) to extract optical and phenotypic metrics—VI, TF, and SF—from UAV hyperspectral and ultra-high-resolution RGB images to investigate the correlation between rapeseed biomass and these metrics at different growth stages. (2) to estimate rapeseed AGB using DNN, SVR, and RF. The study compares the accuracy of VI, TF, SF, and their various combinations, while also evaluating the performance of the three artificial intelligence approaches in estimating AGB across different growth stages.

## 2 Materials and methods

### 2.1 Experimental design

**Figure 1:**
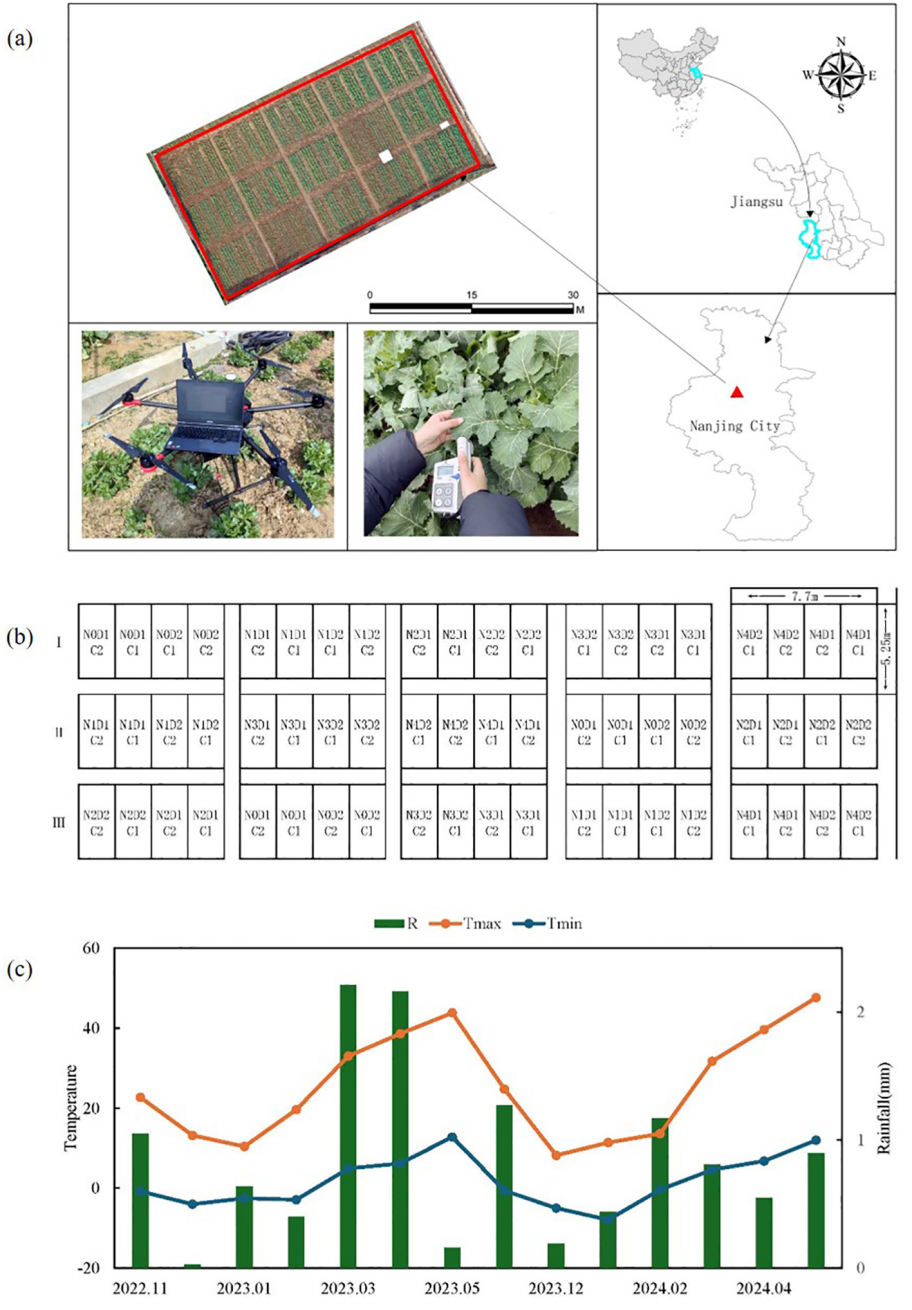
Geographical location of the study area and planting region for rapeseed. **(A)** Sampling area for Measurement data and spectral data collection, **(B)** Field distribution of processing condition. **(C)** Temperature of the crop growing season.

The study was conducted at the Smart Agriculture Research Base of the Jiangsu Academy of Agricultural Sciences, Nanjing, China, located at 32°02′34″N, 118°26′25″E (**Figure 1A**). Two varieties of rapeseed, Zheza 903 (C1) and Ningyou 26 (C2), were used in the study. During the 2022–2023 growing season, nitrogen fertilizer treatments were applied at rates of 0, 90, 180, 270, and 360 kg/ha, labeled as N0, N1, N2, N3, and N4, respectively. Each treatment was replicated three times in a randomized block design on a 600 m² test plot. Phosphorus and potassium fertilizers were applied at rates of 120 kg/ha P_2_O_5_, 180 kg/ha K_2_O, and 15 kg/ha boron. Sowing took place on October 10, 2022, with transplanting on November 5, 2022, at a planting density of 1.125 × 10^5^ plants/hm². Fertilizer was distributed as base fertilizer: wax fertilizer: moss fertilizer in a 5:3:2 ratio, while other cultivation practices followed high-yield field management (**Figure 1B**). During the crop growing season, data on average daily precipitation and minimum temperature were collected in the field (**Figure 1C**). The highest daily average precipitation (53 mm) was recorded in June 2023, while the highest daily temperature occurred in May. The lowest temperature was observed in January.

### 2.2 Data acquisition

#### 2.2.1 Field data acquisition

AGB and plant height (PH) measurement datasets were collected for rapeseed at the seedling stage (December 28, 2022), bolting stage (February 20, 2023), and early blossoming stage (March 2, 2023). Collection of AGB data: To ensure representative sampling, three plants reflecting the overall growth condition were randomly selected from each plot and placed in sealed plastic bags for transportation to the laboratory. Once separated, the stems and leaves were washed with running water and placed in an oven at 105°C for 1 hour, followed by drying at 80°C for more than 48 hours until a stable weight was reached. The stems and leaves were then weighed using a high-precision balance (accuracy 0.001 g), and the total weight of the samples was calculated. AGB was determined based on the population density and the total weight of the samples. Collection of PH data: Four representative rapeseed plants were selected from each plot, and the distance from the base to the tip of the leaf was measured using a ruler. The average of the four measurements was calculated as the PH for the rapeseed in the plot.

#### 2.2.2 Hyperspectral and RGB data collection

A six-rotor UAV (Matrice 600 Pro, DJI, China) equipped with a Resonon PIKA CX imaging spectrometer (Resonon, USA) was used to capture hyperspectral data on sampling days. The hyperspectral sensor covers a spectral range of 400–1000 nm with 2.2 nm spectral resolution across 150 channels. To ensure data consistency, all flights were conducted from the same takeoff point between 12:00 and 13:30 under clear, windless conditions. Each flight followed a consistent route for all growth stages, flying at an altitude of 50 m (with a 6 m transect width) and a speed of 2 m/s. An 80% overlap was maintained in both forward and side directions. Radiometric calibration was performed before each flight using black and white reference panels. Hyperspectral images were acquired at four key growth stages: bare-soil (October 20), seedling, bolting, and early blossoming, with a spatial resolution of 2.5 cm.

RGB images were also captured on the same days and at the same altitude using a quad-rotor UAV (Dajiang Yu2, DJI, China). These images, along with digital surface models (DSM), were processed using Pix4D software. Ground control points (GCPs) were used to align the RGB photos for spatial consistency across growth stages. The images were processed into high-density point clouds, grids, and textures using structure-from-motion (SfM) algorithms, and mosaicked into digital orthophotos for each growth stage.

#### 2.2.3 Hyperspectral data processing

**Figure 2:**
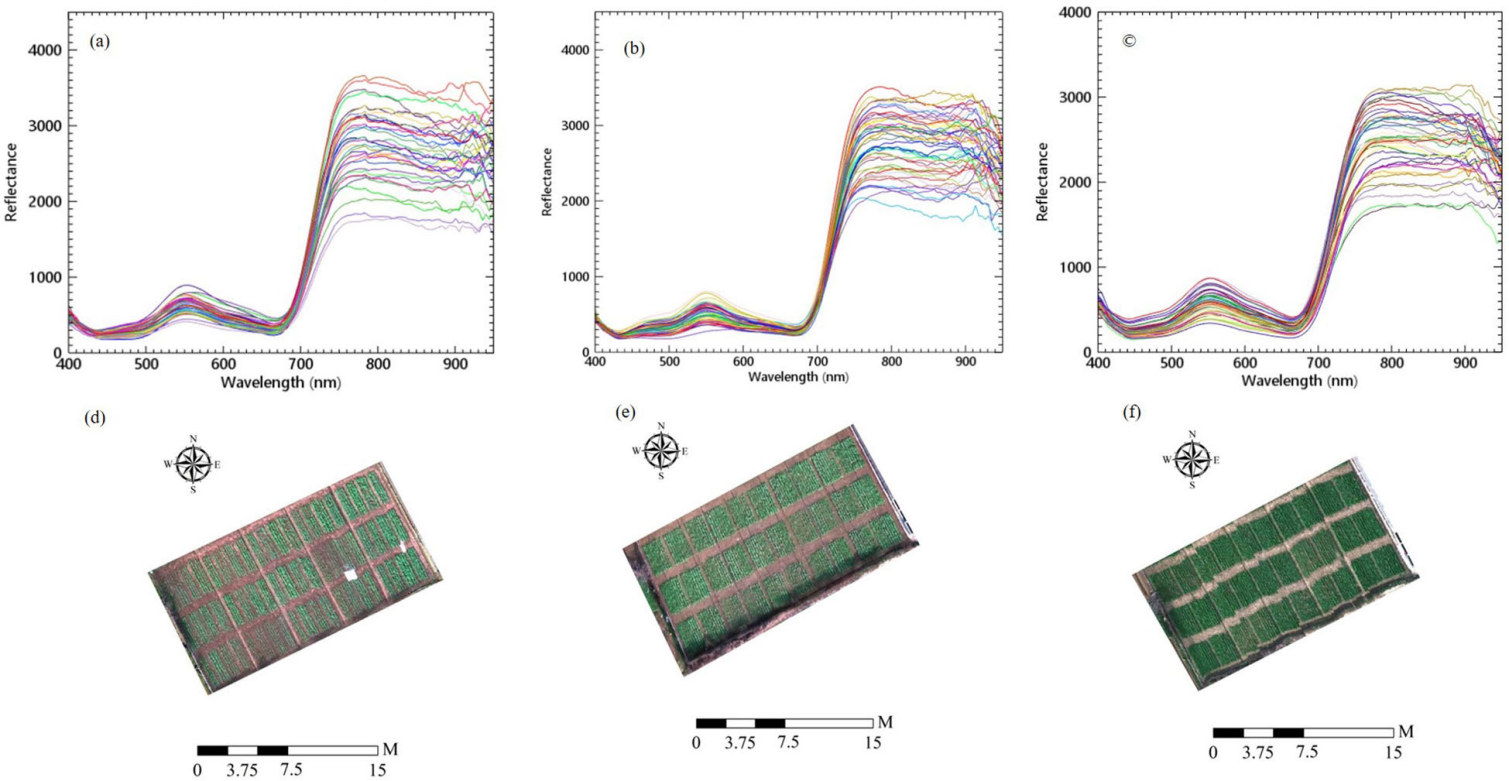
The hyperspectral images and canopy spectral reflectance of rapeseed at **(A, D)** seedling stage, **(B, E)** bolting stage and **(C, F)** early blossoming stage.

The UAV hyperspectral images were preprocessed in three steps: (1) Correction and mosaicking: The data were corrected and mosaicked using Spectronon software (USA) for both radiometric and geographic corrections. (2) Image stitching: ENVI software (Harris Exelis, USA) was used to stitch the images within the specified navigation area, incorporating position data from GCPs to minimize correction error. This resulted in a new hyperspectral image. (3) Extracting rapeseed canopy reflectivity: Rapeseed canopy reflectance curves were extracted from the images. Using ArcGIS software, different maximum area vectors were delineated, and vector data were assigned numbers based on samples. The average spectral reflectance of each region of interest was then extracted using the interactive data language (IDL) in ENVI software. These values were considered the spectral reflectance of the rapeseed canopy in different plots. The images and the average values for each plot were used as the rapeseed canopy spectrum, as illustrated in **Figures 2A–F**.

### 2.3 Optical and phenotypic metrics selection

#### 2.3.1 VI metrics extraction

Canopy spectral information, obtained through optical sensors, is a crucial parameter for monitoring crop growth. The VI is closely linked to the physiological and biochemical characteristics of crops, making it an essential tool for assessing crop development. This index captures the interaction between spectral bands and enhances the response to specific crop properties. Based on previous research, 15 spectral vegetation indices were selected to estimate the AGB of rapeseed. The band calculation tool in ENVI 5.3 software was used to compute these VIs, as detailed in **Table 1**.

**Table 1:** The VI metrics extracted from hyperspectral images.

Vegetation indices	Equation	Reference
NDVI (normalized-difference vegetation index)	(R800 − R680)/(R800 + R680)	(Rouse et al., 1974)
RVI (ratio vegetation index)	R810/R660	(Rouse et al., 1974)
EXG (Excess green vegetation index)	2G-R-B	(Bendig et al., 2013)
EXB (Excess blue vegetation index)	1.4B-G	(Bendig et al., 2013)
NGBVI (Red green blue vegetation index)	(G^2^-BR)/(G^2^+BR)	(Bendig et al., 2013)
NGBDI (Normalized green blue difference index)	(G-B)/(G+B)	(Bendig et al., 2013)
EVI(enhanced vegetation index)	2.5 × (R800 − R670)/(R800 + 6 × R670 − 7.5 × R450 + 1)	(Tao et al., 2020)
SPVI (spectral-polygon vegetation index)	0.4 × [3.7 × (R800 − R670) − 1.2 × |R550 − R670|]	(Tao et al., 2020)
MCARI (modified chlorophyll-absorption ratio index)	((R700 − R670) − 0.2 × (R700 − R550))×(R700/R670)	(Tao et al., 2020)
RNDVI (renormalized-difference vegetation index)	(R800 − R670)/(R800 + R670)1/2	(Tao et al., 2020)
CI_red edge_ (Red edge chlorophyll index)	R810/R690-1	(Gitelson et al., 2003)
VARI (Visible atmospherically resistance index)	(R555 − R680)/(R555 + R680 − R480)	(Gitelson et al., 2002)
SAVI (Soil-adjusted vegetation index)	(1 + 0.5) × (R800 − R670)/(R800 + R670 + 0.5)	(Gnyp et al., 2014)
GNDVI (Green normalized-difference vegetation index)	(R750 − R550)/(R750 + R550)	(Zheng et al., 2019)
SIPI (Structure-insensitive pigment index)	(R800 − R450)/(R800 + R680)	(Li B. et al., 2020)

#### 2.3.2 TF metrics extraction

The Gray Level Co-occurrence Matrix (GLCM) is one of the most widely used methods for texture extraction, originally proposed by Haralick (1973). GLCMs became popular due to their ability to maintain rotational invariance, capture multi-scale features, and allow for low-complexity calculations (Haralick et al., 1973). In this study, three texture metrics—Data Range (DR), Variation (VAR), and Entropy (ENT)—were extracted from UAV RGB bands. The selected window size effectively captures variations in spatial information among the rapeseed plants within the experimental plot. A window that is too small can increase computational complexity and the volume of calculations, while a window that is too large may result in the loss of detailed texture information (Bai et al., 2021). To address this, an averaging technique that combines the functionality of different window sizes was employed. Through trial and error, texture features were computed using the average values of two window sizes (3 pixels × 3 pixels and 5 pixels × 5 pixels) and four directional orientations (0°, 45°, 90°, and 135°) rotated clockwise along the x-axis. These features have been shown to be effective in quantifying changes in crop canopy structure and estimating AGB (Yue et al., 2018a).

#### 2.3.3 SF metrics extraction

In this study, UAV RGB images of rapeseed were captured to create a base map for the DEM before sowing. Canopy point cloud images were then acquired to construct the DSM at various growth stages of rapeseed. The height model for each growth period was derived by subtracting the DEM from the DSM. Using the statistical toolbox in ArcGIS and the Kriging interpolation algorithm, the average plant height (PH) was extracted from the region of interest for each image (Xu et al., 2022). Plant roughness (PR), a metric that characterizes the irregularities of the canopy surface, was measured using 3D point clouds from UAV RGB images. PR has been shown to have a significant correlation with crop AGB (Herrero-Huerta et al., 2020). The fraction of plant cover (PC) was determined through image classification, where ground objects in the RGB images were categorized as either crops or soil (Maimaitijiang et al., 2019). PC for each image was calculated by dividing the number of cropped pixels by the total number of pixels in the image. Building on previous research, the volume method was applied to estimate crop biomass within a defined spatial range. The plant volume metric (PVM) of rapeseed was calculated as the product of PC and PH, along with the canopy elevation fluctuation rate (CEFR) to describe the relative shape of the canopy, as commonly used in forestry studies (Han et al., 2019). The canopy structure metrics extracted from UAV RGB images—PH, PR, PC, PVM, and CEFR—are defined in **Table 2**.

**Table 2:** The SF metrics extracted from UAV-RGB images.

SF metrics	Equation	Reference
PH (Plant Height)	DSM-DEM	(Xu et al., 2022)
PR (Plant Roughness)	IQR^med^	(Herrero-Huerta et al., 2020)
PC (Plant Cover)	Plant Pixel/total pixels	(Maimaitijiang et al., 2019)
PVM (Plant Volume Metric)	$\sum_{i}^{N}S*PH_{i}$	(Han et al., 2019)
CEFR (Canopy Elevation Fluctuation Rate)	(PH_mean_-PH_10%min_)/(PH_10%max_-PH_10%min_)	(Han et al., 2019)

### 2.4 Model construction method

#### 2.4.1 Model construction

The input layer of the DNN model used in this study consists of four hidden layers with 256, 128, 64, and 32 neurons, respectively (Hu et al., 2024). A ReLU activation function was applied after each hidden layer. To address overfitting, a dropout layer with a 0.2 ratio was added after the first hidden layer. The network was trained using the Adaptive Moment Estimation (ADAM) optimizer, with a maximum of 600 training iterations and a batch size of 256. The initial learning rate was set at 0.001, decreasing by 10% every 100 rounds. For the Random Forest (RF) model, bootstrap sampling was used to create a training dataset, and random decision trees were generated based on the integrated classifier (Niu et al., 2019). The RF model was configured with 80 decision trees (n tree = 80) and a maximum number of variables considered at each split (m try = 4). The final prediction was determined through a majority voting process among the decision trees. Support Vector Regression (SVR) was applied for linear and nonlinear regression tasks (Liu et al., 2023). The training dataset was binary-classified using a kernel function to minimize the distance of all samples from the hyperplane. The sample data were then fitted to generate predictions.

#### 2.4.2 Model evaluation

**Figure 3:**
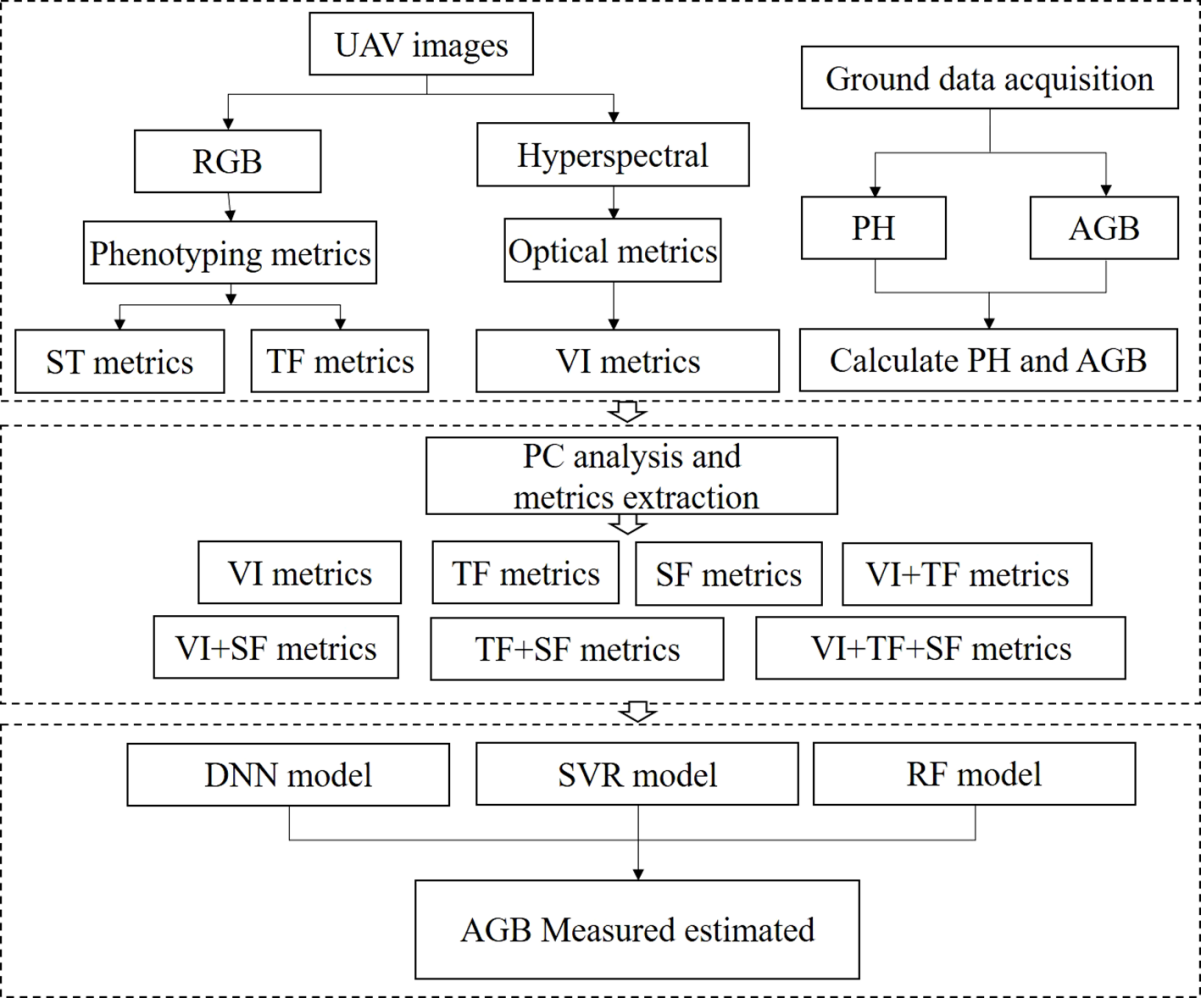
Research workflow.


A total of 60 datasets were collected for each period during the 2022-2023 season. Repeats 1 and 2 were selected as the calibration dataset, while plots from Repeat 3 were used as the validation dataset. The statistical results were presented in **Table 3**. To construct an AGB estimation model for rapeseed across various growth stages, a ten-fold cross-validation approach was employed. Pearson correlation analysis was performed to examine the relationship between features and AGB. The model’s performance and stability were assessed using the coefficient of determination (R²), prediction root mean square error (RMSE), and relative root mean square error (rRMSE). The study workflow was shown in **Figure 3**.

**Table 3:** Descriptive statistics for AGB (kg/ha) and PH (cm) of calibration and validation datasets.

Dataset	Crop parameters	Min	Average	Max	Standard deviation	Coefficient of variation (%)
Calibration	AGB	147.50	1785.60	5573.39	688.56	38.85
	PH	8.90	43.74	110.50	18.26	41.75
Validation	AGB	223.40	1945.23	6061.60	824.47	42.38
	PH	10.10	42.76	105.20	22.74	53.18

(1) $$R^{2}=1-\frac{\sum_{i}{\left(y-y^{\prime }\right)}^{2}}{\sum_{i}{\left(y-\bar{y}\right)}^{2}}$$

(2) $$RMSE=\sqrt{\frac{\sum_{i=1}^{n}{\left(y-y^{\prime }\right)}^{2}}{n}}$$

(3) $$rRMSE=\frac{RMSE}{\bar{y}}$$

Where *y* is the observed value (manual measurement), and $y^{\prime }$ is the predicted value (model extracted value), $\bar{y}$ is the average value, and *n* is the sample size.

## 3 Results

### 3.1 Correlation of metrics and AGB

#### 3.1.1 Statistical analysis of AGB measurements

For the AGB samples, the average value in the calibration dataset was 1785.6 kg/ha, with an overall coefficient of variation of 38.85%, while the validation dataset had a higher coefficient of variation at 42.38% (**Table 3**). The minimum AGB value observed was 147.5 kg/ha, and the maximum was 5573.39 kg/ha. For PH, the average was 43.74 cm in the calibration dataset and 42.76 cm in the validation dataset. The overall coefficient of variation for PH was 41.75%, while the validation dataset exhibited a larger variation of 53.18%. These results indicate that the validation dataset generally showed larger coefficient of variation values compared to the calibration dataset.

#### 3.1.2 Correlation of VI metrics and AGB

**Figure 4:**
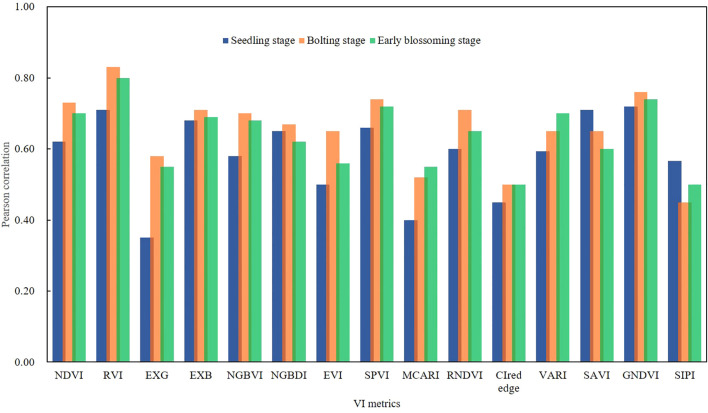
Pearson correlation analysis between VI metrics and AGB for three growth stages.

The correlation between VI metrics and AGB across different growth stages was illustrated in **Figure 4**. The AGB and VI values for the three different growth stages of rapeseed showed strong significance (p< 0.01). The strongest correlation during the seedling stage was observed with the RVI, which had a correlation coefficient of r = 0.82 (p< 0.01). Significant correlations were also noted between AGB and SAVI (r = 0.75, p<0.01) as well as NDVI (r = 0.72, p< 0.01). The results suggested a linear relationship between VI and AGB at all growth stages, although the strength of the correlation decreased as the crop developed. It was important to note that VI tends to saturate when AGB was high, meaning the accuracy of AGB estimation using a single VI may require validation through a more comprehensive estimation model.

#### 3.1.3 Correlation of TF metrics and AGB

**Figure 5:**
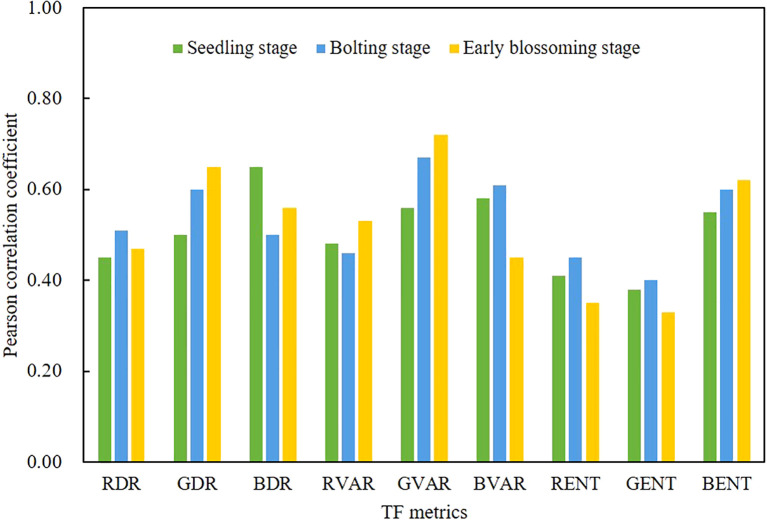
Pearson correlation analysis between TF metrics and AGB for three growth stages.

In this study, the correlation between nine TF metrics and rapeseed AGB across different growth stages was evaluated, as shown in **Figure 5**. The strongest correlation was observed between the GVAR and AGB across all three growth stages, with the average correlation exceeding 0.5 (p< 0.01). The correlations between GDR and BENT were also close to 0.5 (p< 0.05). The results indicate that DR, VAR and ENT metrics exhibit significant variability in relation to AGB, suggesting they fluctuate more throughout the growth stages in response to AGB.

#### 3.1.4 Correlation of SF metrics and AGB

**Figure 6:**
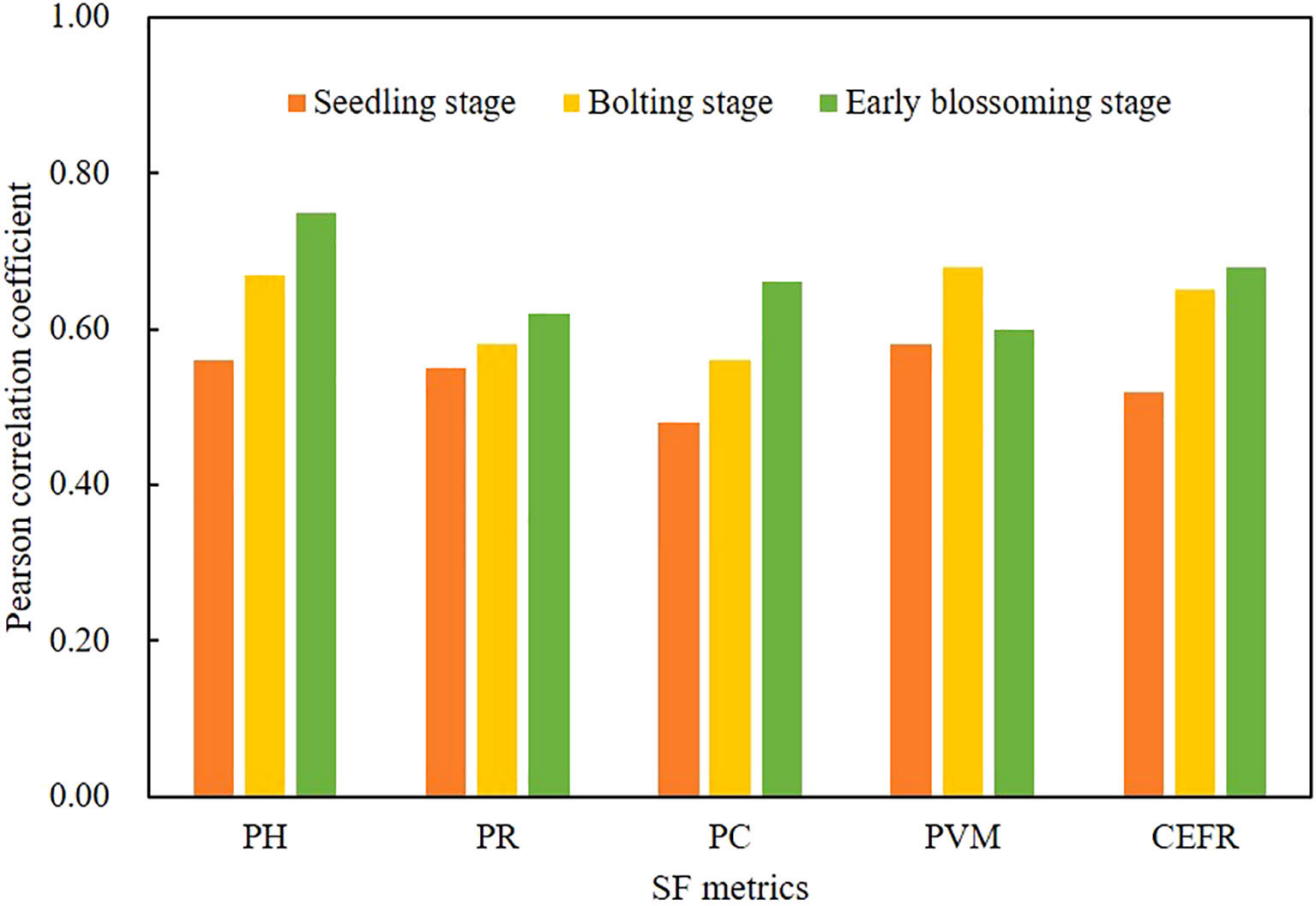
Pearson correlation analysis between SF metrics and AGB for three growth stages.

The correlation between SF metrics and AGB at the seedling, bolting, and Early blossoming stages of rapeseed was shown in **Figure 6**. SF metrics, such as PH, PR, PC, and CEFR, showed significant correlations with AGB across all three growth stages (pp< 0.01). The correlation between these metrics and AGB increased progressively through the growth stages. Notably, the PVM showed a rise in correlation with AGB at first, followed by a decrease in the later growth stages. PH displayed the strongest correlation across all stages, with r values of 0.56 (p< 0.05), 0.67 (p< 0.01), and 0.75 (p< 0.01) at the seedling, bolting, and Early blossoming stages, respectively. These results demonstrate that SF metrics have a linear relationship with AGB at each growth stage.

### 3.2 Construction of AGB estimation models

Based on the results of the correlation analysis, we selected the most significant correlation metrics (r > 0.6) as input variables for the AGB estimation models (**Table 4**). Utilizing both the calibration and validation datasets, we developed rapeseed AGB estimation models through three machine learning algorithms: ANN, SVR, and RF. The regression results, including R², RMSE, and rRMSE, were summarized in **Table 5** and illustrated in **Figure 6**. The findings indicate that, among the estimation models constructed using individual features and the three algorithms, the ANN model combined with VI yielded the best performance. Specifically, the ANN model that employed the combination of VI, TF, and SF achieved the highest R² values of 0.878 and 0.864 for the training and test datasets, respectively. This model also resulted in the lowest RMSE and rRMSE values of 447.02 kg/ha and 0.171, respectively. In contrast, the SVR model using TF exhibited the lowest R² along with the highest RMSE and rRMSE values. The performance ranking of the three algorithms in constructing AGB estimation models was as follows: ANN > RF > SVR.

**Table 4:** Input metrics were selected for algorithms.

Algorithm	Feature	Metrics
DNN, SVR, RF	VI	NDVI, RVI, EXB, NGBDI, RGBVI, EVI, SPVI, RNDVI, VARI, SAVI, GNDVI
	TF	GDR, GVAR, BDR, BVAR, BENT
	SF	PH, PR, PC, PVM, CEFR

**Table 5:** Rapeseed AGB estimates based on different features combination with algorithms.

Features	Algorithm	Calibration			Validation		
		R^2^	RMSE (kg/ha)	rRMSE	R^2^	RMSE (kg/ha)	rRMSE
VI	DNN	**0.765**	**597.27**	**0.235**	**0.705**	**810.51**	**0.316**
	SVR	0.681	683.65	0.272	0.620	951.54	0.351
	RF	0.726	634.51	0.266	0. 661	927.16	0.348
TF	DNN	0.554	823.14	0.347	0.510	1135.84	0.434
	SVR	0.615	767.83	0.312	0.583	1015.21	0.395
	RF	**0.629**	**755.92**	**0.295**	**0.561**	**1054.33**	**0.406**
SF	DNN	0.647	722.16	0.272	0.614	968.78	0.364
	SVR	0.605	774.41	0.311	0.564	1054.65	0.401
	RF	**0.720**	**686.02**	**0.264**	**0.676**	**889.82**	**0.332**
VI+TF	DNN	**0.798**	**550.78**	**0.215**	**0.733**	**803.14**	**0.318**
	SVR	0.732	650.24	0.268	0.703	835.24	0.314
	RF	0.765	540.15	0.235	0.695	848.17	0.326
VI+SF	DNN	**0.823**	**524.95**	**0.201**	**0.785**	**687.82**	**0.264**
	SVR	0.741	645.18	0.244	0.712	788.15	0.292
	RF	0.804	556.47	0.206	0.768	754.52	0.277
TF+SF	DNN	0.755	630.13	0.23	0.712	788.15	0.295
	SVR	0.651	712.54	0.295	0.581	1020.45	0.397
	RF	**0.763**	**638.43**	**0.244**	**0.745**	**762.53**	**0.286**
VI+TF+SF	DNN	**0.878**	**447.02**	**0.171**	**0.864**	**583.85**	**0.224**
	SVR	0.781	563.24	0.227	0.733	775.16	0.295
	RF	0.812	530.15	0.205	0.761	761.58	0.283

The best model under each feature combination is shown in bold.

**Figure 7:**
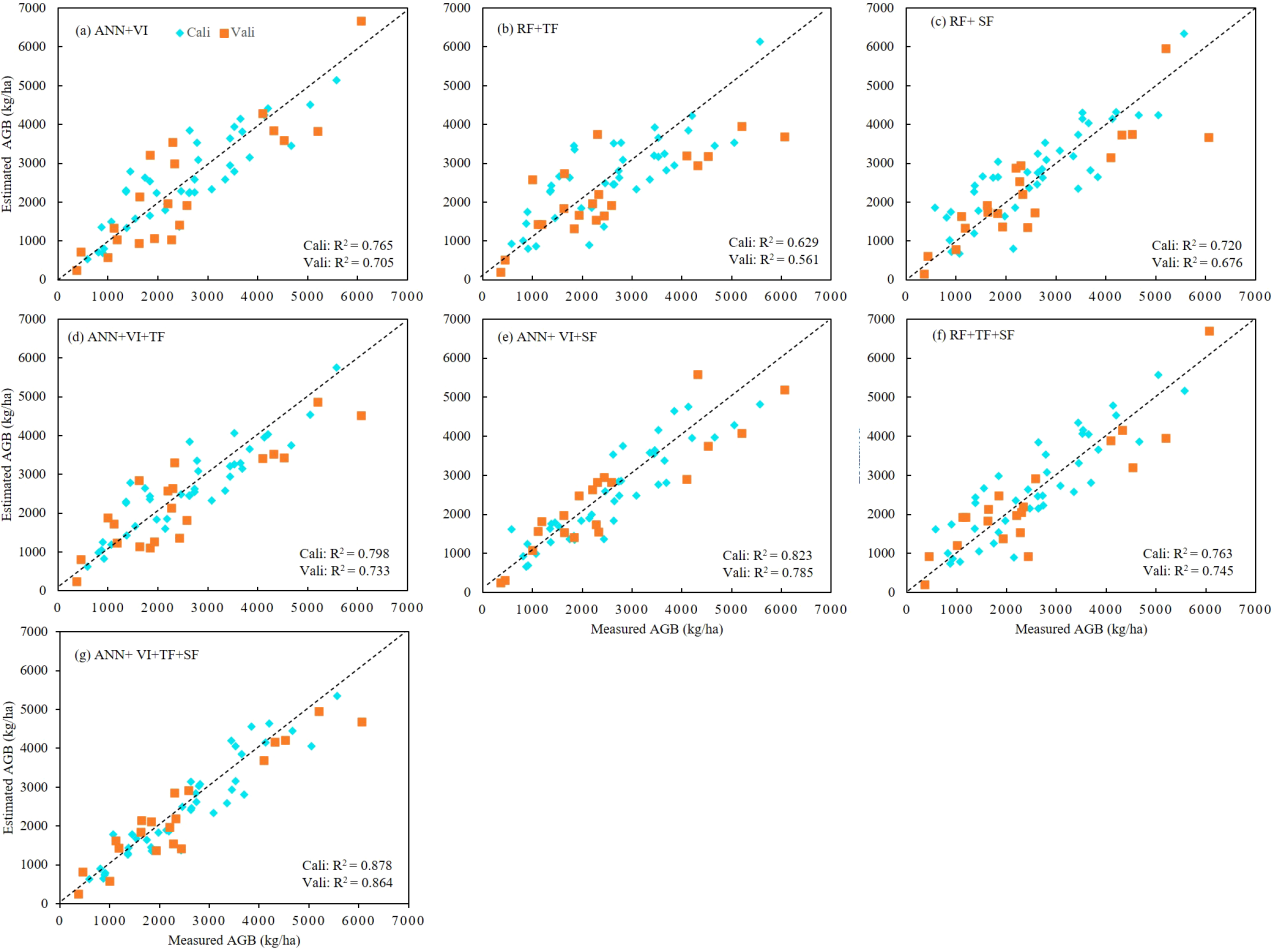
Scatter plots between measured and estimated AGB for calibration and validation datasets of different combination model. **(A)** Combine DNN and VI, **(B)** combine RF and TF, **(C)** combine RF and SF, **(D)** combine DNN and VI+TF, **(E)** combine DNN and VI+SF, **(F)** combine RF and TF+SF, **(G)** combine DNN and VI+TF+SF.

**Figure 7** presents the R² values for models utilizing seven different feature combinations. In four combinations—VI, VI+TF, VI+SF, and VI+TF+SF—the ANN model consistently outperformed both SVR and RF models in terms of R². However, for the TF, SF, and TF+SF combinations, the RF models achieved the highest R² and the lowest RMSE and rRMSE across both training and test datasets. In the VI+TF model, the SVR model recorded the lowest R² and the highest RMSE and rRMSE, while the ANN model ranked second behind RF. In the VI+TF+SF model, the RF achieved the highest R² on the training set, whereas the SVR model displayed the lowest R². However, on the test set, the ANN model produced the highest R². These results confirm that the ANN model, when combined with VI, TF, and SF features, provides the best performance for both training and validation datasets, highlighting the superior capability of the ANN model in estimating rapeseed AGB.

### 3.3 Optimal estimation model of rapeseed at different growth stages

**Figure 8:**
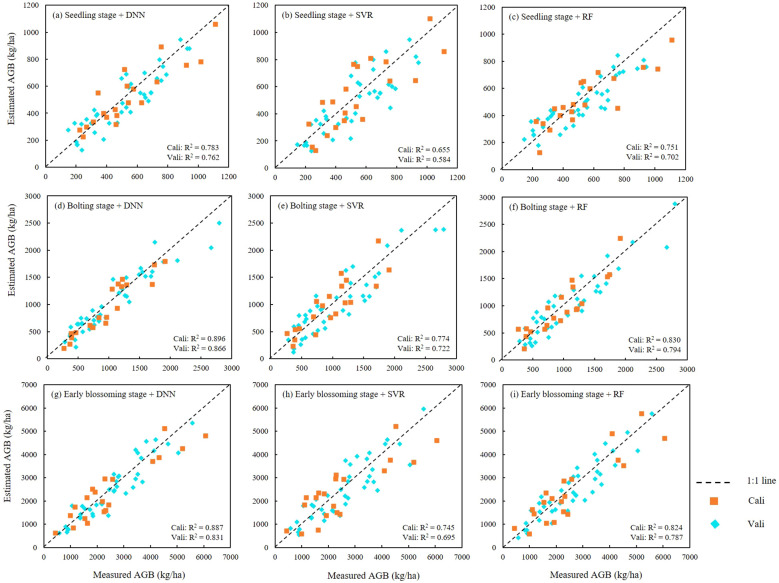
Scatter plots between measured and estimated AGB obtained by DNN, SVR and RF models. **(A–C)** were seedling stage with three algorithms, **(D–F)** were bolting stage with three algorithms, **(G–I)** were Early blossoming stage with three algorithms.

AGB estimation models for rapeseed were constructed for three key growth stages: seedling, bolting, and early blossoming, using three machine learning algorithms: ANN, RF, and SVR. The optimal feature combinations were selected, and the results were presented in **Table 6** and **Figure 8**. Across all growth stages, the ANN model consistently demonstrated the highest accuracy, followed by the RF model, while the SVR model exhibited the lowest accuracy. The high consistency between the training and validation set results further confirms the superior performance of the ANN algorithm. In terms of growth stage comparisons, the ANN model achieved an AGB estimation accuracy of 0.783 during the seedling stage. As the rapeseed developed, the estimation accuracy improved, reaching 0.896 during the bolting stage. However, a slight decrease in accuracy was observed during the early blossoming stage, with a value of 0.878. Similar trends were observed in the RF and SVR models, where estimation accuracy peaked during the bolting stage, outperforming both the seedling and Early blossoming stages. As a result, the AGB maps produced of different periods by optimal estimation model (**Figure 10**).

**Table 6:** Estimated AGB for calibration and validation datasets of three growth stages.

Growth stages	Algorithm	Calibration			Validation		
		R^2^	RMSE (kg/ha)	rRMSE	R^2^	RMSE (kg/ha)	rRMSE
Seedling stage	DNN	**0.783**	**101.67**	**0.195**	**0.762**	**115.64**	**0.213**
	SVR	0.655	129.15	0.251	0.584	155.32	0.284
	RF	0.751	106.93	0.227	0.702	128.53	0.236
Bolting stage	DNN	**0.896**	**193.18**	**0.184**	**0.866**	**169.51**	**0.175**
	SVR	0.774	264.65	0.242	0.722	244.36	0.254
	RF	0.830	229.6	0.214	0.797	209.18	0.216
Early blossoming stage	DNN	**0.878**	**447.01**	**0.177**	**0.831**	**598.77**	**0.238**
	SVR	0.745	609.17	0.236	0.695	804.08	0.319
	RF	0.824	513.32	0.194	0.787	660.14	0.262

The best model under each stage is shown in bold.

### 3.4 Evaluation of variable importance

**Figure 9:**
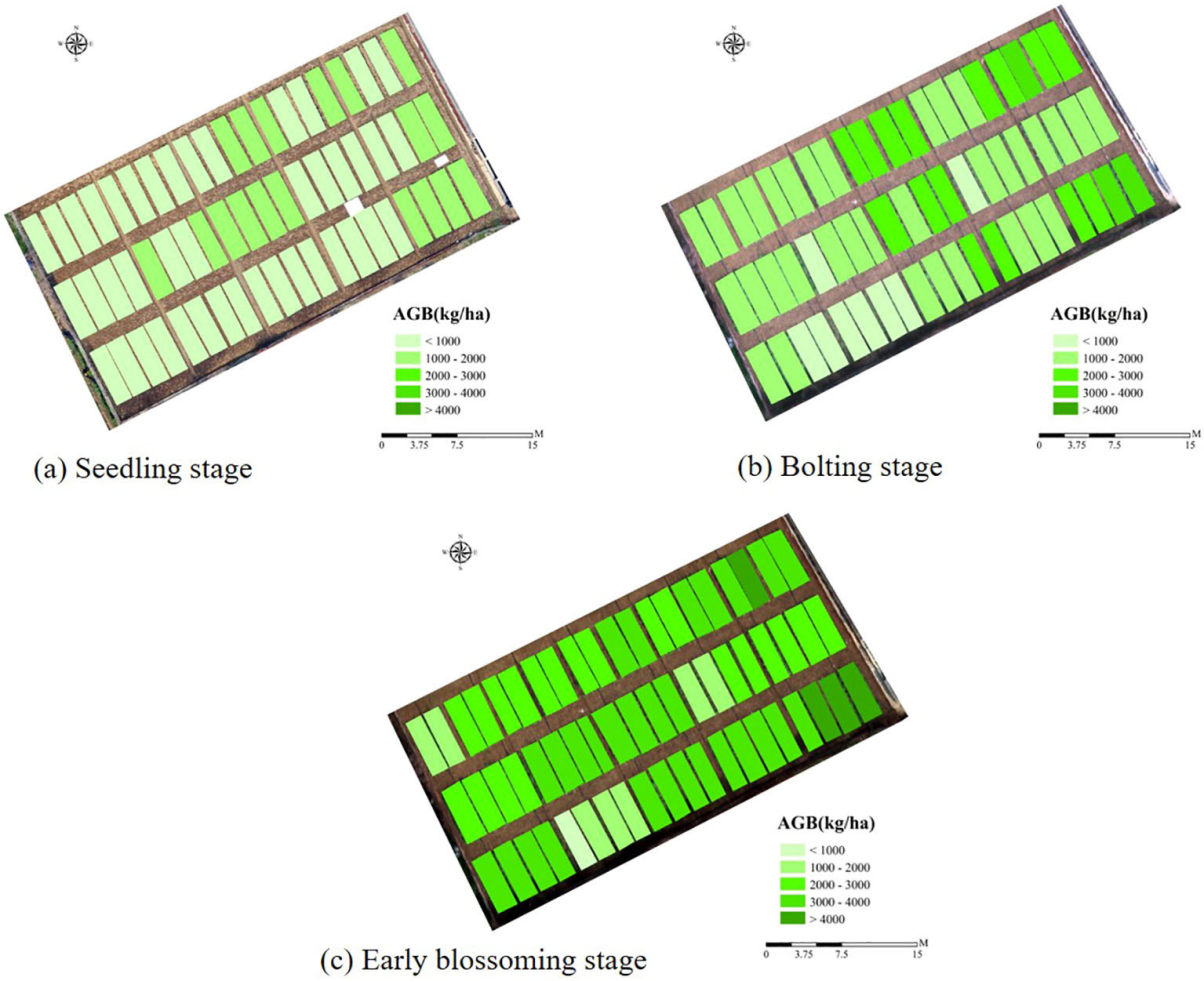
Mapping rapeseed AGB using three metrics and DNN model. **(A)** Seedling stage, **(B)** Bolting stage, **(C)** Early blossoming stage.

To assess the contribution of different input metrics to the estimation models, we applied the RF importance evaluation method. **Figure 9** illustrates the variable importance scores of three rapeseed growth stages. During the seedling stage, the RVI metric exhibited the highest importance, with vegetation indices contributing approximately 50% of the overall importance. However, as the crop progressed to the bolting stage, structural metrics gained prominence, with PH becoming a key factor in AGB estimation. The variable importance in the early blossoming stage closely mirrored the results of the bolting stage, highlighting the continued relevance of structural metrics at later stages of development. Overall, these findings were consistent with the performance of the AGB estimation models, reflecting the shift in the relative importance of vegetation and structural parameters as rapeseed matures.

**Figure 10:**
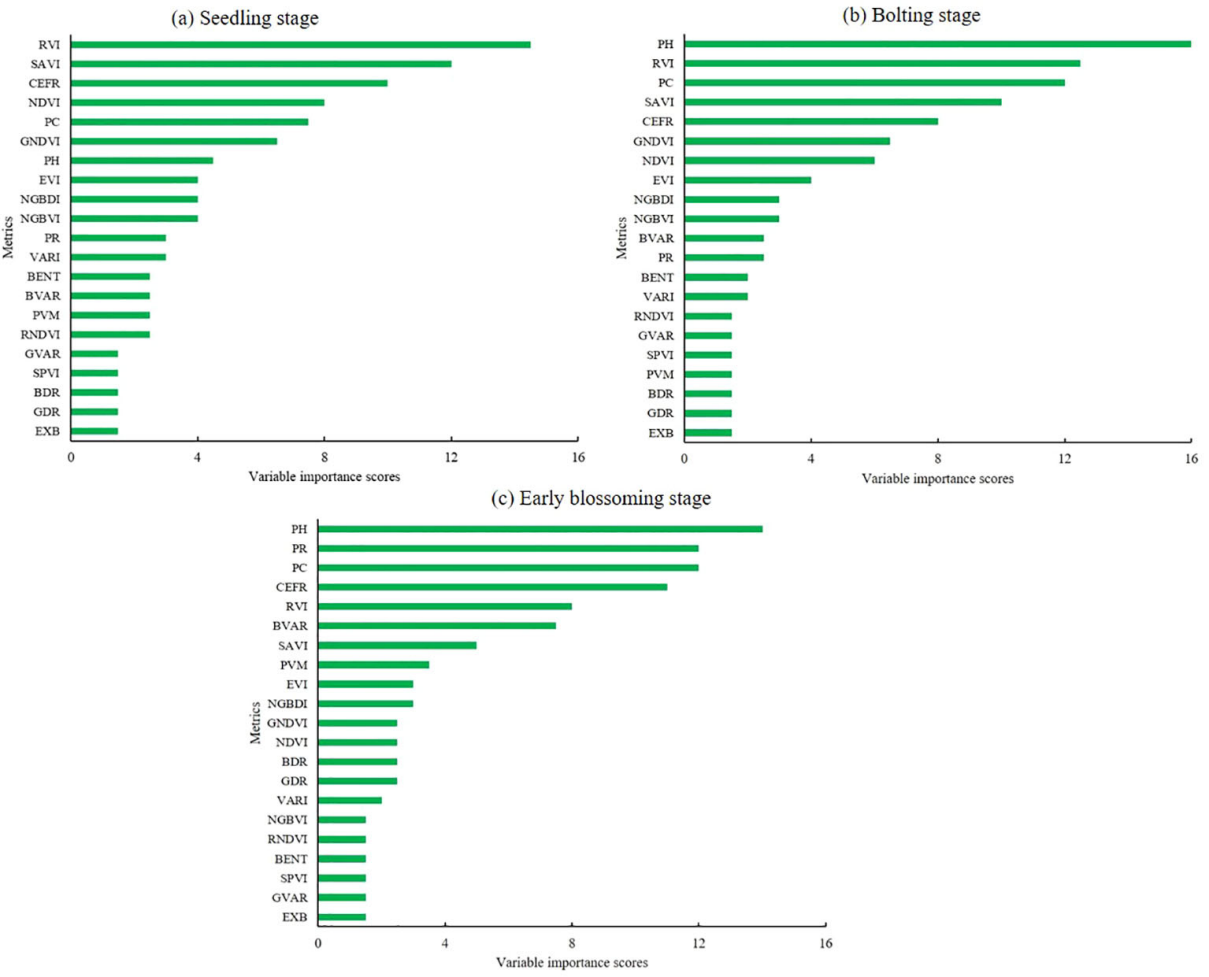
The variable importance scores of three growth stages. **(A)** Seedling stage, **(B)** Bolting stage, **(C)** Early blossoming stage.

## 4 Discussion

### 4.1 The correlation of metrics and rapeseed AGB

This study assessed the effectiveness of combining spectral, textural, and structural features derived from UAV-based hyperspectral and RGB imagery to enhance the accuracy of AGB estimation in winter rapeseed. Previous research has demonstrated the high accuracy of UAV-based methods for estimating plant biomass (Liu et al., 2022, 2023; Niu et al., 2019). Consistent with these studies, our analysis found significant correlations between rapeseed AGB and the spectral, textural, and structural parameters extracted from UAV imagery. However, we observed that the stability of these correlations varied across different growth stages. While individual features, such as vegetation indices, texture metrics, and structural characteristics, were strongly correlated with AGB, their predictive power fluctuated as the crop developed. Notably, a combined approach leveraging the strengths of spectral, textural, and structural features offers considerable potential for achieving more accurate and consistent AGB estimates throughout rapeseed’s growth cycle.

The correlation analysis between VI, TF, and SF with rapeseed AGB shows distinct trends across different growth stages. VI tend to saturate as spectral parameters stabilize, resulting in peak correlations during the bolting stage, following the seedling stage. These correlations gradually weaken as the crop enters the early blossoming stage (Liu et al., 2023). In contrast, TF and SF demonstrate increasing complexity with crop growth, reflecting the canopy’s development. This complexity aligns with the rise in aboveground biomass, suggesting these features may provide complementary insights into rapeseed AGB estimation.

### 4.2 Estimation performance of various metrics

Previous studies have indicated that texture metrics can outperform VI in predicting above-ground biomass (AGB), though much of this research has focused on forests, with relatively limited applications in crop biomass estimation (Basyuni et al., 2023; Nichol and Sarker, 2011; Xu et al., 2024). In our study, we found that among individual feature types, SF produced more accurate AGB estimates than TF. Moreover, the integration of VI+TF+SF led to significant improvements in AGB estimation for winter rapeseed by reducing RMSE compared to models that relied solely on VI, TF, or SF. Contrary to earlier findings, our results did not show that texture metrics alone outperformed VI in estimating AGB. Instead, our study highlights that the combination of VI and SF yielded better AGB estimates than the combination of VI and TF (Schumacher et al., 2016; Xu et al., 2022, 2024).

Comparing the estimation capabilities of different features at various growth stages of rapeseed, we found that structural parameters yield higher accuracy in biomass estimation as the crop matures. This aligns with the importance evaluation, which shows that SF like PH, PR, and PC become increasingly significant as the crop develops. Integrating multiple feature types—VI, TF, and SF—offers a more comprehensive approach to estimating AGB, as each capture unique and complementary information about crop growth. The highest estimation accuracy was observed during the bolting stage, providing crucial insights for guiding fertilization decisions during this key phase of rapeseed development.

### 4.3 Advantages of model estimation

Our findings demonstrate the effectiveness of machine learning models—specifically RF, SVR, and DNN—in estimating rapeseed AGB. Across the three growth stages, the DNN model consistently achieved the highest R² (0.896) and the lowest RMSE (193.18kg/ha) and rRMSE (0.184) (**Table 6**). Although the overall performance of the RF model was slightly lower than that of the DNN model, it demonstrated superior accuracy when TF and SF were combined as input variables. In this case, the RF model outperformed the DNN model in terms of R² and rRMSE on the test set, indicating that RF may be more adept at processing texture and structural data. The strong performance of the DNN model is likely due to its deep iterative layers, which allow it to capture complex patterns in the data, highlighting its potential for biomass estimation. However, the RF model’s ability to handle TF and SF features effectively suggests that it is particularly well-suited for integrating these types of data. The evaluation of feature importance also revealed that the relative contribution of different input parameters significantly impacts model performance, further explaining the variations in accuracy among the algorithms.

The estimation results indicate that the inclusion of structural parameters significantly improved the performance of the estimation models, suggesting that the enhancement in estimation accuracy is related to the addition of key estimation factors (such as PH, PR, and PC). Among the three algorithms used to construct the estimation models, the DNN model demonstrated a clear advantage across all three growth stages, while the RF model performed better during the bolting and early flowering stages. In contrast, the SVR model showed weaker estimation performance in all stages. This suggests that the DNN algorithm exhibits good practical applicability for estimating rapeseed AGB at different growth stages.

### 4.4 Research outlook

The process from data collection to model development requires meticulous attention to detail and thorough analysis. Improving the quality of data obtained from UAV imagery, ground observations, and modeling techniques is crucial, as these datasets can be prone to errors. Standardizing these procedures is essential for ensuring consistency and accuracy.

While this study presents an effective approach by integrating three feature types for estimating rapeseed AGB, several challenges remain. Data accuracy in hyperspectral acquisition and preprocessing must be carefully managed, and the mixed pixel problem due to resolution constraints may impact estimation performance. Additionally, the comparison of biomass under different nitrogen treatments is a valuable area of research that warrants further investigation. Future studies could extend the scope by exploring rapeseed biomass estimation over multiple years to evaluate the applicability and transferability of the developed models, thus enhancing their generalizability. Furthermore, delving deeper into the biophysical properties of plants and identifying potential error sources will be crucial for further refining estimation accuracy.

## 5 Conclusions

This study explored the potential of UAV hyperspectral and RGB imagery for estimating crop biomass by developing a multi-feature estimation model that incorporates VI, TF, and SF. The performance of these features in estimating rapeseed AGB across different growth stages was thoroughly evaluated. The RF importance evaluation method was used to assess the contribution of different input parameters to the estimation model. The results indicated that both DNN and RF outperformed SVR when using individual features for AGB estimation. Additionally, the DNN model surpassed the RF model in accuracy when feature combinations (VI, TF, and SF) were applied, achieving the best estimation performance across all growth stages. Furthermore, the DNN model (R² = 0.878, RMSE = 447.02 kg/ha) with the combined features outperformed both the RF (R² = 0.812, RMSE = 530.15 kg/ha) and SVR (R² = 0.781, RMSE = 563.24 kg/ha) models. Based on the variable importance analysis, the RVI index emerged as the most significant, while PH was identified as a key phenotypic metric in AGB estimation. These findings demonstrate that integrating hyperspectral and RGB data with advanced artificial intelligence models, particularly DNN, provides an effective approach for estimating rapeseed AGB. The estimation model incorporating VI, TF, and SF showed higher accuracy in estimating rapeseed AGB compared to models using individual feature sets. Among the growth stages, the bolting stage yielded slightly higher estimation accuracy than the seedling and early blossoming stages. The combination of VI, TF, and SF metrics offers significant improvements in biomass estimation accuracy, highlighting the potential of UAV-based multi-feature modeling in precision agriculture.

## Data Availability

The raw data supporting the conclusions of this article will be made available by the authors, without undue reservation.
